# Complexity based measures of postural stability provide novel evidence of functional decline in fragile X premutation carriers

**DOI:** 10.1186/s12984-019-0560-6

**Published:** 2019-07-12

**Authors:** Clodagh O’Keeffe, Laura P. Taboada, Niamh Feerick, Louise Gallagher, Timothy Lynch, Richard B. Reilly

**Affiliations:** 1 Trinity Centre for Biomedical Engineering, Trinity College, The University of Dublin, 152 - 160 Pearse St, Dublin 2, Ireland; 2 School of Medicine, Trinity College, The University of Dublin, Dublin, Ireland; 3 Department of Psychiatry, School of Medicine, Trinity College Dublin, Dublin, Ireland; 4 The Dublin Neurological Institute at the Mater Misericordiae University Hospital, Dublin, Ireland; 5 Centre for Neuroscience, Conway Institute, University College Dublin, Dublin, Ireland; 6 Mater Misericordiae University Hospital, Dublin, Ireland; 7 School of Engineering, Trinity College, The University of Dublin, Dublin, Ireland

**Keywords:** Multiscale entropy, Dual-task, Balance, FMR1 premutation, FXTAS, Cognitive-motor interference

## Abstract

**Background:**

Fragile X Associated Tremor/Ataxia Syndrome (FXTAS) is a neurodegenerative movement disorder characterized by tremor, ataxic gait, and balance issues resulting from a premutation of the Fragile X Mental Retardation 1 (FMR1) gene. No biomarkers have yet been identified to allow early diagnosis of FXTAS, however, recent studies have reported subtle issues in the stability of younger premutation carriers, before disease onset. This study investigates the efficacy of multiscale entropy analysis (MSE) in detecting early changes in the motor system of premutation carriers without FXTAS.

**Methods:**

Sway complexity of 12 female Premutation carriers and 15 healthy Controls were measured under four conditions: eyes open, closed, and two dual-task conditions. A Sustained Attention Response Task (SART) and a working memory based N-Back task were employed to increase cognitive load while standing on the forceplate. A Complexity Index (Ci) was calculated for anterior-posterior (AP) and mediolateral (ML) sway. Independent t-tests were used to assess between-group differences and Oneway repeated measures ANOVA were used to assess within group differences with Bonferroni corrections to adjust for multiple comparisons.

**Results:**

Group performances were comparable with eyes open and closed conditions. The Carrier group’s Ci was consistent across tasks and conditions while the Control group’s AP Ci increased significantly during the cognitive dual-task (*p* = 0.001). There was also a strong correlation between CGG repeat length and complexity for the Carrier group (*p* = 0.004).

**Significance:**

Increased sway complexity is believed to stem from reallocation of attention to facilitate the increased cognitive demands of dual-tasks. Carriers’ complexity did not change during dual-tasks, possibly indicating capacity interference and inefficient division of attention. Lower sway complexity in carriers suggests diminished adaptive capacity under stress as well as degradation of motor functioning. Therefore, sway complexity may be a useful tool in identifying early functional decline in FMR1 premutation carriers as well as monitoring progression towards disease onset.

## Introduction

Fragile X Associated Tremor/Ataxia Syndrome (FXTAS) is a late-onset neurodegenerative movement disorder characterized by progressive cognitive decline, intention tremor, ataxic gait as well as impaired postural control and balance, which subsequently leads to increased risk of falls. Although highly penetrant, with 45% of males and 16% of female FMR1 premutation carriers over the age of 50 developing the disorder, as of yet, it is unknown why some carriers develop FXTAS while others do not [1, 2]. Unambiguous diagnosis of FXTAS and monitoring of disease progression relies on accurate assessment of movement and stability.

FXTAS has been associated with neurophysiological changes such as reduced cerebellar volume, and aberrant structural connectivity among the superior and middle cerebellar peduncles [3–5]. Such regions are heavily involved in the cortico-ponto-cerebellar feedback loop, imperative for adaptive postural control [6]. Emerging evidence has shown carriers both male and female carriers with FXTAS and those younger than the typical age of onset to exhibited subtle changes in postural stability [7] and that such changes were mediated by reduced cerebellar volume and disruption in vulnerable cerebellar circuits [8]. However, due to the increased prevalence and greater symptom severity of motor symptoms observed in male carriers, the motor phenotype associated with female carriers requires further exploration.

Emerging evidence has found, however, that impairments in postural control were exacerbated when conducting a concurrent cognitive task [9–11]. for example, female Premutation carriers without FXTAS, exhibit larger sway area during concurrent working memory tasks [9]. A similar study found not only that a dual-task resulted in greater attentional cost during the cognitive tasks but that the dual-task effect on the motor task was strongly predicted by working memory capacity [10, 11]. This suggests that postural control may be contingent on the attentional load of the cognitive task combined with individual differences in executive control. As postural control involves constant maintenance by higher order attentional systems and multisensory integration such as vestibular, visual, and somatosensory inputs [12, 13], changes or impairments any of these faculties, may alter stability. Manipulating these faculties through dual cognitive/motor tasks offers a sensitive approach to evaluate cognitive, attentional demands on motor control in FMR1 premutation.

Postural control is regulated through multisensory integration, various cognitive functions, and musculoskeletal outputs, communicating through spinal and supraspinal circuits, each operating over different timescales [12, 14]. Therefore the postural control system is intrinsically non-linear. The influence of each of these cognitive and sensory inputs and muscular outputs are constantly reattuned and reweighted, to maintain stability [14, 15]. These continuous adjustments and changes in control strategy generate seemingly spontaneous fluctuations in postural sway. Multiscale Entropy analysis (MSE) employs Sample Entropy (SampleEN) to quantify the degree of irregularity or ‘complexity’ in a time series across multiple scales. Postural sway complexity has been found to reflect an individual’s capability to respond and adapt to balance perturbations and stressors, such that greater postural sway complexity reflects a greater ability to adapt to such unexpected stressors [16, 17].

Measuring this physiological complexity of postural sway will reveal the integrity of the postural control system, and better characterize the impact of the FMR1 premutation on balance and stability, subsequently enabling the detection of early changes in the motor system of premutation carriers. A loss or reduction in complexity is believed to stem from the degradation of the physiological systems underpinning postural control or deterioration in the efficiently of communication between these systems [18].

The aim of the current study was, therefore, to examine postural control in a cohort of young female Fragile X premutation carriers by probing sensory and cognitive deficits previously reported to be impaired in younger carriers. This may then detect the subtle differences in postural stability and provide insight into the discriminating power of Entropy-based measures in order to identify parameter(s) which are sensitive to postural instability in the Fragile X premutation carriers. Previous studies have shown the degree of postural sway complexity to be largely independent of classical parameters [19]. Therefore, it was hypothesised that non-linear measures of postural sway will more accurately reflect the integrity of the postural control system in younger Fragile X Premutation Carriers. Specifically, it was hypothesised that Premutation Carriers would exhibit reduced sway complexity compared to the Control group and that the increased cognitive load associated with the dual-task paradigm would exacerbate differences in sway complexity in carriers.

## Methods

### Participants

Twelve female Fragile X Premutation Carriers (37–45 years) were recruited through advocacy groups and social media. Inclusion criteria for carriers included a positive genetic test for the FMR1 gene premutation. Fifteen Controls (28–47 years) were recruited through similar means. See Table 1 for summary demographics. All subjects had normal or corrected to normal vision and no intellectual disability as assessed using the Weschler Abbreviated Scale of Intelligence (FSIQ> 70) [20]. Subjects were excluded from the Control group if a first-degree relative had a neurological condition. No subject reported any transient, neurological or musculoskeletal problem that may affect balance. Informed consent was obtained from all participants and all procedures followed were in accordance with ethical requirements of the Tallaght Hospital and St. James’s Hospital Joint Research Ethics Committee as well as the School of Medicine Ethics Committee, Trinity College Dublin.

**Table 1:** Summary of descriptive data

	12 PM carriers Mean (SD)		15 Healthy Controls Mean (SD)		*P-*value
Age	41.2	(3.12)	37.1	(5.5)	0.012*
Education (years)	17.7	(3.7)	19.2	(2.5)	0.214
FSIQ^a^	109.6	(16.3)	118.7	(14.81)	0.14
Verbal comprehension^a^	114	(15.28)	123	(12.6.)	0.119
Perceptual reasoning^a^	102.4	(16.24)	114.2	(19.66)	0.108
Weight (Kg)	75.1	(20.77)	65.8	(9.51)	0.136
CGG repeat length	83.1	(8.12)	–	–	–
SART					
Correct (%)	89.44	(7.4)	89.84	(10.0)	0.909
No. Of Errors	6.45	(4.89)	6.6	(7.5)	0.663
N-back^b^					
Correct (%)	91.36	(11.3)	90.44	(10.26)	0.826
No. Of Errors	3.17	(3.43)	2.6	(3.22)	0.956

*p < 0.05

^a^Parameters derived from the WASI-II

^b^*n* = 11 for PM carriers

### Procedure

Subjects stood, without shoes, in a comfortable bilateral stance near the center of the force platform with arms by their sides. To ensure consistency between trials, an outline of their feet was made on the platform surface. An eye-level visual reference was presented on a screen at a distance of 1.5 m. Subjects were advised to be aware of their balance and to stand as still as possible for the duration of the trial. Three 90-s trials were conducted with eyes open (EO) and three 90-s trials were carried out with eyes closed (EC). Two dual-task conditions were completed while standing: a three-minute letter variant N-back task [21] and a number variant Sustained Attention Response Task (SART) [22]. During the N-back task, participants were told to respond when the number on screen was the same as the number presented immediately previous (1-Back) while the SART involved displaying the numbers 0 to 9 on screen while participants responded for each number except the number three. The duration of this task was 5 min and subjects were reminded to stay as still as possible during both dual-tasks. Both tasks required subjects to provide a response using a handheld touchpad. The order of the cognitive tasks was randomised, with EO and EC trials repeated between each cognitive task. Subjects were encouraged to take a break between trials to avoid fatigue. Force plate data were acquired after the completion of each task trial to avoid any detectable end effects. The first and last 15 s of each EO and EC trial were removed to avoid initial transient, anticipatory and impact effects. Although the N-back and SART had a duration of 3 and 5 min respectively, only the second 60 s of each cognitive task was used for analyses, to ensure each time series was of equal length as well as to mitigate practice and fatigue-related effects.

### Data processing and analysis

Postural data was collected using a Biosignals Plux Forceplate (Plux Wireless Biosignals, S.A. Portugal) at a sampling rate of 1000 Hz. The reaction force generated from the application of pressure to each of the four cells of the forceplate was converted to cartesian coordinates, each relating to directional subcomponents (Y-axis = Anterior-Posterior(AP); X-axis = Medio-Lateral(ML)).

### Classical parameters of sway

Postural sway was characterized using Classical sway parameters such as path length, sway area (as measured by a 95% confidence ellipse), Velocity_AP_ and Velocity_ML_. Data collected from the forceplate were filtered using a 4th order low-pass Butterworth filter with a cut off of 10 Hz and downsampled to 50 Hz before sway parameters were calculated [23]. All analyses were carried out using Matlab_R2016a (The Mathworks Inc., Natick, MA).

### Multiscale entropy analysis

To quantify postural complexity, both the AP and ML subcomponents of the time series were subjected to MSE analysis. Prior to MSE analysis, the forceplate data was downsampled to 250 Hz and detrended using empirical mode decomposition (EMD) [24]. The lowest frequency suitable for inclusion in the analyses was determined using the algorithmic procedure outlined by Gow et al. [25]. High-frequency noise (above 20 Hz) and low-frequency trends (below 0.8 Hz) were filtered to ensure a sufficient number of physiologically meaningful dynamic patterns were available for analysis [16, 26]. To capture system dynamics over multiple time scales, a ‘course graining’ procedure was used to divide the time series into non-overlapping windows of length equal to a scaling factor *Ƭ*, ranging from 1 to 40 data points. As the second 60 s epoch of each task was used for analyses, at the largest scale, each course-grained series contained 375 data points (15000 points/40). Based on previous recommendations, SampleEn was computed for each course grained time series, where m = 2 and *r* = 15% [16].

**Fig. 1:**
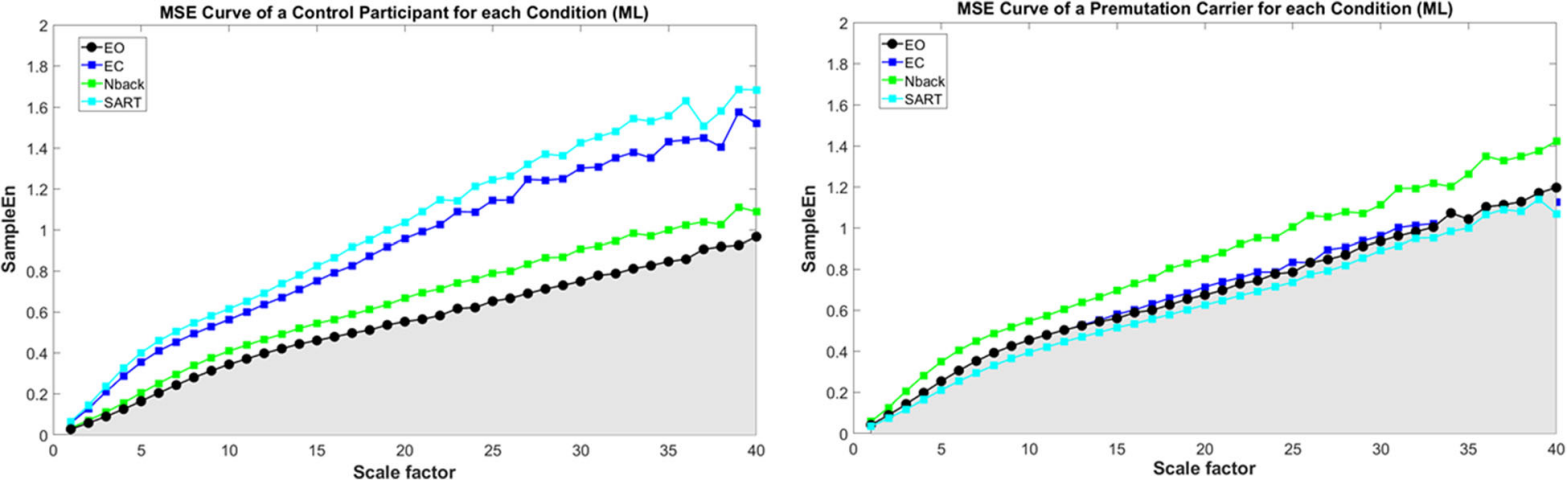
Representative MSE curves generated from AP sway time-series of a single Control subject (left) and Premutation Carrier (right). The shaded area represents the complexity index (Ci), defined as the area under the MSE curve where higher curves indicate greater complexity

To construct MSE curves, SampleEn was plotted as a function of time-scale *Ƭ*. The postural sway complexity Index (Ci) was measured as the area under the MSE curve, such that higher SampleEn values over multiple time series results in larger area, which reflects greater complexity (see Fig. 1). All data processing was carried out using Matlab R2016b (Mathworks, Natick, MA; [27]) and the Waveform database V.0.9.10 (Wfdb toolbox, [28]).

### Statistical analysis

A Shapiro-Wilk test of normality determined that the distribution of the data. Mann-Whitney U tests were used to compare mean performances of classical parameters between both groups and the Wilcoxon signed-rank test was used to conduct within-group comparisons, with Bonferroni corrections to adjust for multiple comparisons. Independent t-tests were used to assess between-group differences and Oneway repeated measures ANOVA were used to assess within group differences with Bonferroni corrections to adjust for multiple comparisons, thus reducing the significance level to *p* < 0.007. Pearsons correlations were used to assess the relationship between age and repeat length, and outcome variables.

## Results

Both groups were comparable in terms of demographic data. The Premutation group was significantly older than the Control group, (*p* = 0.012). Data from only 10 Premutation Carriers were available for the N-back task due to an error in recording. There was no significant difference between Premutation Carriers and Controls in terms of their performance during either SART or N-back task (Table 1).

### Classical parameters

There were no differences between groups in path length, sway area, or velocity in either ML or AP direction, during the EO and EC conditions (*p* > 0.05). Both groups were also comparable in terms of classical sway parameter during both the SART and N-back tasks (*p* > 0.05 for all comparisons). Within-group analysis also did not reveal a significant change in sway area or velocity between the baseline EO condition and either dual-task conditions (*p* > 0.05 for both). Path Length was found to increase during the EC condition of the Control group (*p* = 0.001) and the Premutation group (*p* = 0.005). There was also a strong correlation between the error rate during the SART task and sway velocity in the ML direction for the Carrier group, whereby velocity increased with the number of errors committed (*r* = 0.672 *p* = 0.01).

### Complexity index

An independent sample t-test revealed no difference in Ci between groups either with EO or EC in the AP or ML direction (*p* > .05). Ci of both groups were also also comparable during the SART task (AP: *t* (25)= − 1.297, *p* = .184, *d* = .67; ML: *t* (25) =0.601, *p* = .553, *d = .23*). Although the mean N-back AP Ci of the control group was greater than that of the PM group, this difference did not reach significance, (*t* (25)= − 1.774, *p* = .089, *d = .72*) See Table 2.

**Table 2:** Complexity indices of premutation carriers and control subjects across conditions

	EO Mean (STD)		EC Mean (STD)		N-Back Mean (STD)		SART Mean (STD)	
	AP	ML	AP	ML	AP	ML	AP	ML
PM	26.2 (6.7)	24.6 (5.71)	27.6 (4.78)	28.7 (6.78)	29.532 (4.8)	25.377 (5.68)	27.31 (5.13)	30.04 (5.79)
Ctrl	24.8 (8.02)	26.43 (4.63)	28.5 (4.4)	27.7 (6.25)	33.9 (7.96)	29.33 (8.21)	30.88 (8.33)	28.24 (3.96)
*p-*value	0.64	0.38	0.62	0.71	0.09	0.19	0.18	0.55

*PM* Premtuation carriers, *Ctrl* Controls, *STD* Standard deviation

*p < 0.05

**Fig. 2:**
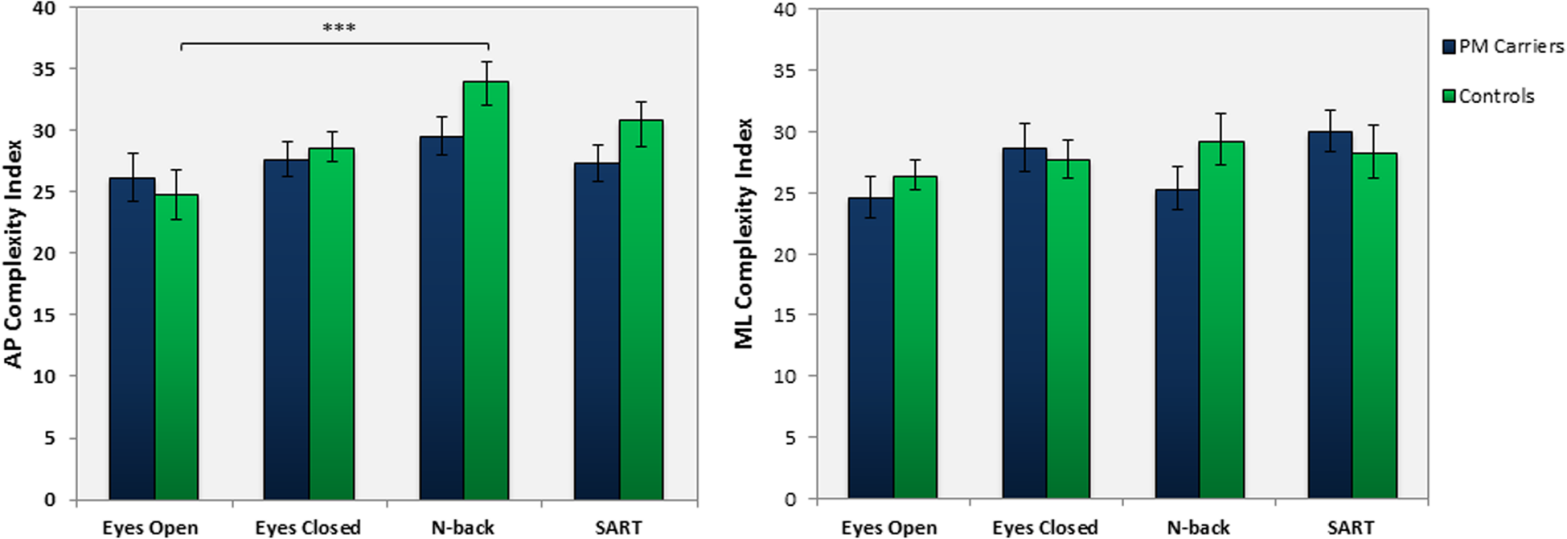
Complexity Index of sway for Premutation carriers and Controls. Complexity index of anterior-posterior sway during eyes open (EO) and eyes closed (EC) conditions, SART, and N-back dual tasks (left). Complexity index of postural sway in the mediolateral direction under all four conditions (right). All data expressed as mean ± standard error. ***p < 0.001. *Brackets* indicate significant differences between conditions

Within-group analysis did not reveal a change in AP nor ML postural stability complexity between the EO and EC condition for Premutation Carriers or Controls. The ML complexity index of Premutation Carriers remained consistent across each task (*F* (3, 27)= 2.1,*p* = .124, ηp^2^ = .19). Although the Carriers’ ML Ci increased during the N-back task compared to EO (*p* = .029, *d* = 0.41), this did not survive correction for multiple comparisons. The Control group, however, did exhibit a change in complexity across conditions an increase in AP Ci during the N-Back task (*p* = .001, *d* = 0.9). See Fig. 2. The ML Ci of the Control group did not differ significantly from the EO condition (*F*(3,42) = .509, *p* = .67, ηp^2^ = .1).

### Correlations

Pearson’s Correlations were carried out to assess the relationship between performance on the cognitive dual-tasks and the complexity of postural sway. During the SART task, there was a strong positive correlation between AP Ci of carriers and the number correct responses (*r* = 0.71, *p* = 0.014) No such correlation observed in the Control group or ML Ci for Premutation Carriers. There was no relationship found between the number of correct responses or error rate during the N-back task and Ci of Premutation Carriers in either AP or ML direction (*p* > 0.05 for all correlations).

**Fig. 3:**
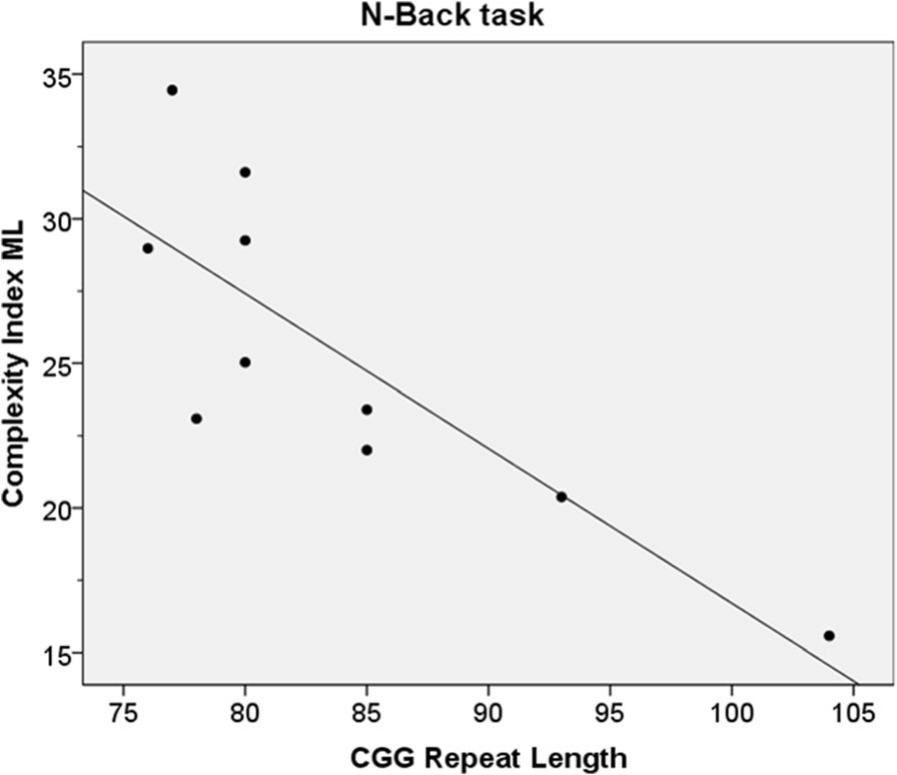
Correlation between Complexity Index of ML sway and CGG repeat length. Mediolateral Complexity index was strongly correlated with CGG repeat length of the Premutation Group during the N-back task (*r* = 0.71, *p* = 0.01**)

Pearson’s Correlations revealed a strong inverse correlation between CGG repeat length and ML Ci during the N-back task, where Complexity decreased as repeat length increased (*r* = − 0.819*, p* = 0.004.) See Fig. 3. There was no correlation found between age and Ci of either the Premutation or the Control group.

## Discussion

This study employs multiscale entropy-based measures of postural complexity in FMR1 Premutation Carriers, across specifically designed balance tasks, including manipulated sensory and cognitive conditions. The current study aimed to employ a unique analysis of postural control in young female Fragile X Premutation Carriers to detect subtle differences and trends in postural stability which may distinguish younger Carriers from Control subjects. The findings of this study indicate differences between groups in the complexity index of postural sway, particularly with regard to dual-task interference, which were not readily apparent using only classical sway parameters. Additionally, the study also reaffirms the efficacy of the dual-task paradigm in distinguishing age or genetically modulated changes in stability of Premutation Carriers which may be useful in predicting the onset of FXTAS.

Stability is based on complex interactions between the individual, the environment, and the task at hand [14]. During the eyes closed condition, reducing the visual information available to subjects did not reveal a change in sway complexity when compared with standing with eyes open. This suggests removing visual cues of stability did not produce a striking effect on the complexity of postural control systems, which reflects the findings of previous studies involving healthy subjects [29]. Classical sway parameters did reveal an increase in path length of both groups during the EC conditions. Taken together, the results of both Complexity based measures and the classical parameters suggest that although balance was challenged by reduced visual input, both groups were equally capable of adapting to this increased demand on the postural control systems.

Classical measures of sway did not exhibit changes in balance or stability during either of the dual-task conditions. This is contrary to emerging reports on postural control in female Fragile X Premutation Carriers [9]. This may be due to the smaller sample size involved in this study or perhaps classical parameters were not sufficiently sensitive to detect subtle subclinical changes in stability. While MSE analysis revealed that the complexity of the Premutation group was not significantly different from that of Controls, complexity of Carriers’ sway was less during the working memory based dual-task conditions. Such findings reported in other studies in similar cohorts, which have associated lower sway complexity with diminished capacity to adapt to environmental and cognitive stressors [16, 30], as well as being linked to degradation of motor functionality including slow gait, frailty, and increased falls [31], all of which are common symptoms of FXTAS [32].

Increasing cognitive load with a dual-task paradigm adds stress to the postural control system. Performance of a secondary cognitive task causes a shift or division of attention between multiple competing demands, which resulted in a change in the postural complexity in the Control group during the dual-task condition. This interpretation of change in complexity during dual-task is in line with the “facilitatory-control” view of dual-task complexity [33]. This states that an increase in randomness of postural sway may stem from the reallocation of attention to facilitate the supraspinal cortical tasks, such as letter recall or sustaining attention as in the case of this study. The Ci of the Control group increased during the working memory task, perhaps indicating the recruitment of additional attentional/postural resources to cope with the increase in cognitive demand during the dual-task [16]. The Premutation group did not, however, show a change in complexity compared to baseline measures, suggesting a reduction in capacity to adapt to the increase in cognitive demand during the dual-task. However, as there was no difference between groups in terms of cognitive performance, perhaps, indicating the carrier group prioritised performance of the cognitive task over postural performance. This may suggest an inability to divide attention efficiently between both tasks. Similar dual-task related impairments were reported in relation to gait parameters [34], balance and postural control [9] in younger carriers while carrying out memory-based verbal fluency task. Such results may be indicative of capacity interference in Premutation Carriers, whereby several competing demands contend for limited attentional resources. Similar dual-task methodologies in other cohorts have shown that a secondary cognitive task diverts attention from the primary motor task, compounding impairments in already weakened systems [13].

The differences in postural sway complexity, particularly during dual-tasks may, therefore, serve as a marker by which to distinguish Premutation Carriers from unaffected Control subjects. However, large-scale longitudinal studies would be necessary to determine whether these changes in complexity are the result of stable neurodevelopmental changes caused by the neurotoxic effect of the FMR1 premutation or are indicative of progressive neurodegeneration. Further exploration would be required to establish the efficacy of postural sway complexity as a biomarker for FXTAS.

The results of this study did not reveal age-modulated effect on complexity scores of Premutation Carriers during either single or dual-task conditions. This is not consistent with previous findings that carriers experience worsening of symptoms with age [1, 35, 36]. This is also not in line with the complexity theory of aging, which states that disease and age diminish the quality and/or quantity of inputs that regulate system behavior, subsequently reducing system functionality [30]. These antithetical findings may be due to the modest sample size employed in this study and the relatively narrow age range of the participants who took part.

Our results do, however, indicate a link between CGG repeat length and postural complexity. This is in line with previous findings which suggest phenotype severity increases with repeat expansions [9, 32, 37]. Higher CGG repeat lengths are associated with increased levels of FMR1 mRNA, the proposed cause of neuropathology seen in FXTAS [38]. Larger repeat expansions have also been linked with a reduction in FMRP levels, a protein vital for neuronal maturation and plasticity. It is believed that decreased levels of FMRP found in Premutation Carriers, impacts cognitive development, therefore, underlining many of the cognitive issues associated with the premutation. The severity of the FMR1 phenotype has also been linked with activation ratio in female carriers (the ratio of cells with the normal allele present on the active X chromosome [39]. Higher activation ratios have been found to have protective or compensatory effects on both cognitive and motor symptoms in carriers. Future research would benefit from taking activation ration data into account as this may shed additional light on such findings.

## Conclusion

To conclude, the current study revealed novel evidence of differences in the physiological complexity of postural stability in female Premutation Carriers. Most importantly, the preliminary findings of this study demonstrate the feasibility and subtlety of multiscale entropy analysis in identifying distinguishing features of functional decline in younger Premutation Carriers, which may not be distinguishable by classical sway parameters. Given the primary motor features of FXTAS include balance and gait issues, early detection of these motor changes will be vital for the detecting carriers who may be at risk of FXTAS, and monitoring progression before the onset of the disorder.

## Data Availability

The datasets used and analysed during the current study are available from the corresponding author on request.

## Abbreviations

Ci: Complexity Index
EC: Eyes closed
EMD: Empirical mode decomposition
EO: Eyes open
FXTAS: Fragile X Associated Tremor/Ataxia Syndrome
IMF: Intrinsic mode function
MSE: Multiscale entropy analysis
SampleEn: Sample Entropy
SART: Sustained attention response task
